# Efficacy and safety of Chinese patent medicine in the adjuvant treatment of prostate cancer: A Bayesian network meta-analysis

**DOI:** 10.1097/MD.0000000000037180

**Published:** 2024-03-22

**Authors:** Shiheng Wang, Fengxia Zhang, Jianfeng Liu, Peijie Qin

**Affiliations:** a Institute for History of Medicine and Medical Literature, China Academy of Chinese Medical Sciences, Dongcheng District, Beijing, China.

**Keywords:** Chinese patent medicine, efficacy, prostate cancer, safety, systematic review Bayesian network meta-analysis

## Abstract

**Background:**

Prostate cancer is the most common cancer in men. In China, traditional Chinese medicine is used to treat prostate cancer. However, there is a lack of evidence for differences in the effectiveness and safety of different Chinese patent medicines. Therefore, we conducted this Network Meta-analysis to investigate the efficacy and safety of different Chinese patent medicines in the treatment of prostate cancer.

**Methods:**

We systematically search PubMed, Web of Science, Embase, Cochrane library, CNKI database, VIP database, wanfang database, and SinoMed Randomized controlled trials of Chinese patent medicines for the treatment of prostate cancer sores included in the database were retrieved until June 1, 2023. The included studies were assessed for risk of bias using Cochrane randomized controlled trial Bias risk Assessment tool. The main outcome indicators were Efficacy, Prostate Specific Antigen, and adverse reaction. Since different courses of treatment were used in the included studies, we used Bayesian mesh meta-regression to investigate the effects of treatment courses on efficacy and safety.

**Results:**

Twenty-seven articles were included, involving 1885 patients. Including 9 kinds of Chinese patent medicine. The results of Network Meta-analysis show that: ① efficacy: compared with androgen antagonists, Bruceolic oil emulsion (relative risk = 1.70, 95% credibility interval [CI] (1.30, 2.29)), Compound Kushen injection (relative risk = 1.39, 95%CI (1.19, 1.70)) had significant advantages. There was no significant difference among all Chinese patent medicines (*P* > .05). The top 3 Chinese patent medicines were Bruceolic oil emulsion, Zhibodihuang pill, Compound Kushen injection. ② Prostate specific antigen: compared with androgen antagonists, Bruceolic oil emulsion (mean difference [MD] = −10.4, 95%CI [−17.6, −3.21]), Compound Kushen injection (MD = −4.46, 95%CI [−8.80, −1.70]), Shenfu injection (MD = −14.7, 95%CI [−23.4, −6.01]) had significant advantages. The top 3 Chinese patent medicines were Shenfu injection, Bruceolic oil emulsion, Compound Kushen injection. adverse reaction: compared with androgen antagonists, there was no significant difference among all PCM (*P* > .05).

**Conclusion:**

Compared with androgen antagonists, Chinese patent medicine has significant difference in effectiveness. The effect of Chinese patent medicine is little affected by the course of treatment and dose. From comprehensive analysis, Bruceolic oil emulsion combined with androgen antagonist is the best intervention measures.

## 1. Introduction

Prostate cancer is an epithelial malignancy of the prostate gland with high morbidity and mortality. According to statistics, in 2020, there will be 1414,259 new cases of prostate cancer worldwide, accounting for 7.3% of the total number of new cancer patients, ranking second in the rate of new cancer. With 375,304 deaths, 3.8% of all cancer deaths, it is the fifth most common cancer death rate in men.^[1]^ With the aging of the population, the incidence and mortality of prostate cancer have increased significantly. By 2040, prostate cancer is projected to increase to nearly 2.3 million new cases and 740,000 deaths globally due to population growth and aging alone.^[2,3]^ Prostate cancer is a major public health problem worldwide.

At present, the treatment methods for prostate cancer include surgery, chemotherapy, androgen antagonists, etc, but there are some complications and adverse reaction, which may reduce the quality of life of patients, and may even develop castration-resistant prostate cancer.^[4]^ In China, traditional Chinese medicine has been tried to use in the adjuvant treatment of prostate cancer, which can reduce the toxic side effects of conventional treatment, improve the quality of life and prolong the survival time,^[5]^ and has attracted increasing attention from clinical researchers. Chinese patent medicine has the advantages of carrying, convenient taking and high patient compliance.

Current Chinese patent medicine for prostate cancer treatment include Qilan capsule,Juzao pill, Zeguilong Shuang Capsule, Kushen injection, Compound Xuanju Capsule, etc. Many clinical trials of Chinese patent medicines for prostate cancer have been published; however, the differences in efficacy and safety between them are unclear. Therefore, by using Bayesian network meta-analysis, this study studied the effectiveness and safety of different Chinese patent medicines, providing more reference value for clinical application and research.

## 2. Data and methods

### 2.1. Research registration

This study was conducted in accordance with reporting guidelines for systematic Review and network meta-analysis (PRISMA-NMA) and is prospectually registered with PROSPERO (ID: CRD42023451261).

### 2.2. Ethical approval

This study is a systematic review, the research object is literature, and does not involve ethical issues.

### 2.3. Literature search strategy

Randomized controlled trials of Chinese patent medicines for prostate cancer were retrieved from CNKI, Wanfang Database, VIP database, Chinese Biomedical Literature Service System, PubMed, Web of Science, Embase, and Cochrane library up to June 1, 2023. The search strategy was constructed according to PICOS tool: (1) population: prostate cancer patients; (2) intervention measures: Chinese patent medicine; (3) control group: conventional treatment (androgen antagonist); (4) outcome indicators: outcome indicators of prostate cancer treatment; (5) study type: randomized controlled trial. See Appendix S1, Supplemental Digital Content, http://links.lww.com/MD/L588, for a detailed search strategy.

### 2.4. Inclusion criteria and exclusion criteria

#### 2.4.1. Inclusion criteria.

(1) Populations: patients diagnosed with prostate cancer according to the diagnostic criteria, or patients explicitly described in the article as having prostate cancer.(2) Interventions: Chinese patent medicine or combined with conventional therapy.(3) Control: Conventional therapy. In order to minimize heterogeneity, conventional therapy included in the study was consistent.(4) Outcomes: Main outcomes: Efficacy; prostate-specific antigen (PSA); An adverse reaction. Secondary outcome measures: Quality of life: According to the Karnofsky performance scale, scores were scored before and after treatment, where an increase of more than 10 points compared with that before treatment was regarded as improvement, a decrease of more than 10 points was regarded as decline, and a change of <10 points was regarded as stability, and an increase or stability of score was regarded as improvement of quality of life.^[6]^ (5) Study type: randomized controlled trial of Chinese patent medicine in the treatment of prostate cancer.

### 2.5. Exclusion criteria

(1) Republished literatures were included according to the principle of the most complete data and the most recent publication year. (2) Summary, abstract, basic test, etc. (3) Literature with incomplete data that cannot be analyzed. (4) The intervention did not correspond to the literature. (5) The full text of the literature cannot be obtained.

### 2.6. Literature screening

Two researchers (Wang SH and Zhang FX) imported the retrieved literature into EndnoteX9 software, and proposed the republished literature by automatically labeling the duplicate literature and manually identifying the duplicate literature. For the remaining literature, unqualified literature was deleted by reading the title and abstract, and the full text of the compliant literature was downloaded. Final reading of the full text for further screening to identify the original studies that fit our systematic review. When the information extracted by the 2 people is inconsistent, they first discuss with each other to solve the problem. If they still cannot solve the problem, they consult the third researcher (Qin PJ).

### 2.7. Data extraction

Two researchers (Wang SH and Zhang FX) designed the data extraction table according to the information needed by the research and extracted it independently. The contents include: ① Basic information: title, author, year, study type, diagnostic criteria, intervention measures, course of treatment, outcome indicators; ② demographic characteristics: sample size, age, sex; ③ methodological information: random method, assignment hiding scheme, blind method, etc. When the information extracted by the 2 people is inconsistent, they first discuss with each other to solve the problem. If they still cannot solve the problem, they consult the third researcher (Qin PJ).

### 2.8. Bias risk assessment

Two researchers (Wang SH and Zhang FX) used the Cochrane randomized Controlled Trial Bias Risk Assessment tool^[7]^ to assess the risk of bias. The evaluation tool contained the following 7 items: random sequence generation, assignment hiding, blinding of subjects and intervention providers, blinding of outcome evaluators, incomplete outcome data, selective outcome reporting, and other sources of bias, with each item assessing the outcome as low bias, high bias, or unclear. Visual presentation of bias risk assessment results using Revman5.4.

### 2.9. Statistical analysis

Counting and continuous variables are calculated with relative risk [RR], mean difference [MD], and 95% confidence intervals (CI). Since there are certainly potential differences between studies, we chose a random effects model for our analysis rather than a fixed effects model. We use the gemtc package of R4.2.0 software for NMA polymerization and analysis using Markov chain Monte Carlo simulation chains in a Bayes-based framework according to the PRISMA NMA specification. We will use the node method to quantify and prove the consistency between indirect and direct comparisons, calculated by instructions in the Stata software, if the *P*-value is >.05. Consistency check passed.

Stata16.0 software was used to present and describe the network diagram of different exercise interventions. In the generated network diagram, each node represents a different motor intervention and a different control condition, and the lines connecting the nodes represent a direct positive comparison between the interventions. The size of each node and the width of the connecting lines are proportional to the number of studies.

The efficacy of each index was sequenced to obtain the surface under the cumulative ran-king (SUCRA), and the probability sequenced was plotted as a graph. SUCRA is expressed as a percentage, with a larger percentage indicating that the intervention is more effective, and a value of 0 indicating that the intervention is completely ineffective. While SUCRA can be usefully reexpressed as a percentage of the effectiveness or acceptability of exercise interventions, these scores should be interpreted with caution unless there are actual clinically meaningful differences between interventions.

## 3. Results

### 3.1. Literature screening results

**Figure 1.**
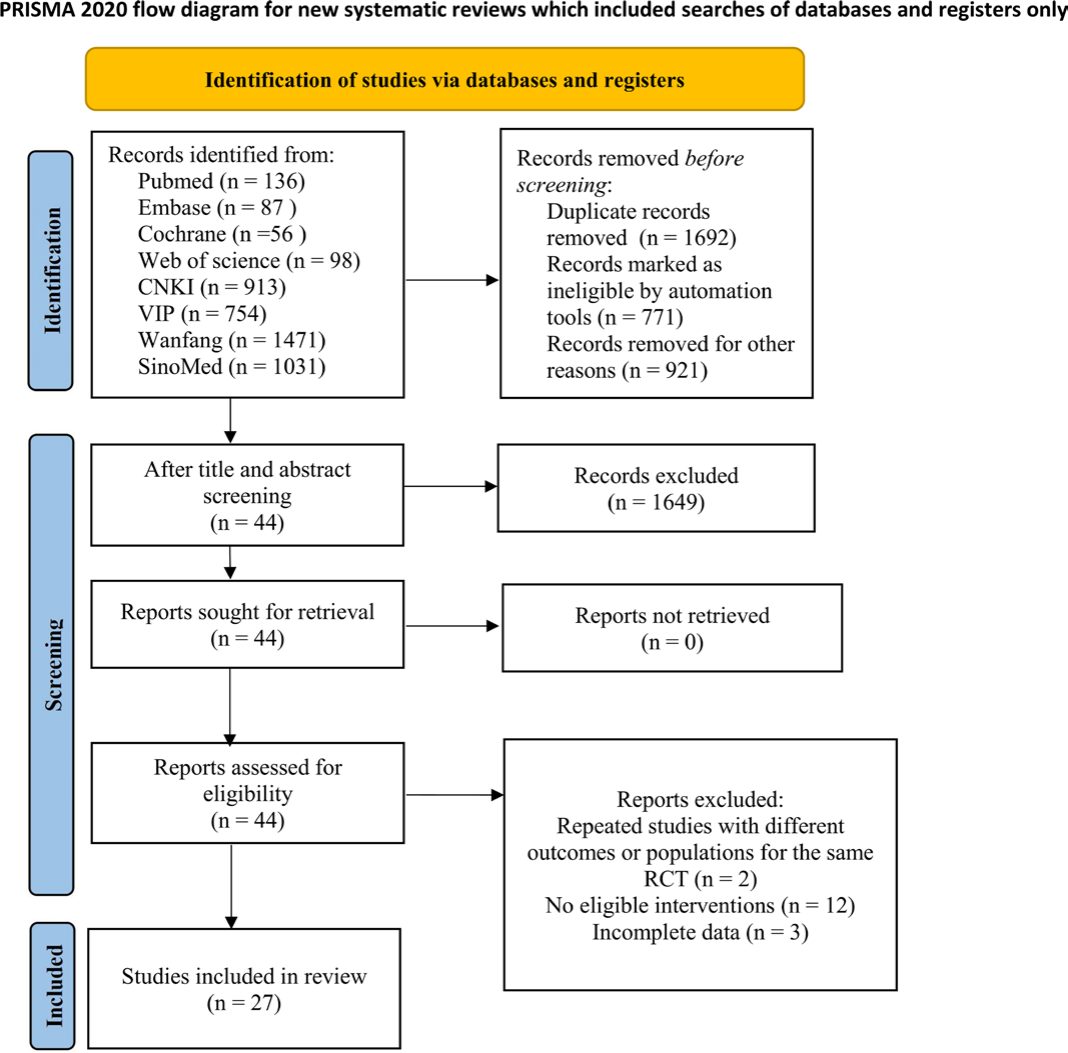
Literature screening process.

A total of 4346 literatures were screened, 1692 duplicate literatures were excluded, 1649 were excluded from reading abstracts and titles, 17 were excluded from reading full texts, and 27 studies were included. Figure 1 is the literature screening flow chart.

### 3.2. Basic features of the included literature

A total of 27 articles were included, involving 1885 patients (959 patients in the experimental group and 926 patients in the control group). They were published in Chinese from 2007 to 2021. The intervention measures in the trial group included 9 proprietary Chinese medicines, Shenfu injection, Shenqifuzheng injection, Compound Kushen injection, Jianpiyishen granule, Juzao pill, Niaochangshu capsule, Qilan capsule, Brucea oil emulsion, Zhibodihuang pill. The control group was treated with androgen antagonists (including leuprelin acetate microspheres, Flutamide, zoredronic acid injection, goserrelin acetate, Bicalutamide, etc). The course of treatment ranges from 8 to 24 weeks (Table 1).

**Table 1:** Basic characteristics of the included literatures.

Author	Year	Sample size		Age		Intervening measure		Course	Dose	Outcomes
		I	C	I	C	I	C			
Zhang ZN^[8]^	2021	40	39	57.48 ± 7.59	57.31 ± 8.33	SFI+AA	AA	12w	50 mL	PSA, KPS, adverse reaction
You SJ^[30]^	2013	21	16	–	–	SQFZI+AA	AA	8w	250 mL	Efficacy ,PSA
Wang YB^[31]^	2021	37	37	63.62 ± 3.78	66.84 ± 4.17	SQFZI+AA	AA	24w	250 mL	Efficacy, PSA, adverse reaction
Yin J^[32]^	2014	21	21	82.9	83.2	SQFZI+AA	AA	12w	15 mL	Efficacy
Liu H^[16]^	2016	35	35	–	–	CKSI+AA	AA	12w	15 mL	Efficacy, PSA, KPS
Gao Z^[18]^	2011	25	25	71.76	74.04	CKSI+AA	AA	8w	20 mL	PSA, KPS
Zhang XW^[33]^	20l3	20	20	–	–	CKSI+AA	AA	12w	20 mL	Efficacy
Zhang XW^[34]^	20l3	25	25			CKSI+AA	AA	12w	30 mL	Efficacy, PSA
Gong WY^[9]^	2019	75	75	57 ± 5	57 ± 5	CKSI+AA	AA	24w	15 mL	Efficacy, PSA
Shi GJ^[35]^	2014	25	16	59–78	58–80	CKSI+AA	AA	12w	30 mL	PSA, adverse reaction ,KPS
Hu KB^[36]^	2014	33	33	–	–	CKSI+AA	AA	12w	5 mL	Efficacy
Duan GC^[20]^	2019	33	28	57.1 ± 9.7	55.3 ± 6.9	CKSI+AA	AA	8w	30 mL	Efficacy, PSA, adverse reaction
Tu R^[10]^	2015	18	18	73.07 ± 7.21	67.20 ± 12.02	CKSI+AA	AA	24w	10 g	Efficacy, PSA
Chen L^[23]^	2017	75	75	65.4 ± 14.7	64.7 ± 13.7	CKSI+AA	AA	8w	3 g	PSA, KPS, Efficacy, adverse reaction
Li J^[19]^	2015	25	25	–	–	CKSI+AA	AA	12w	30 g	PSA, adverse reaction
Liu H^[17]^	2012	45	45	68.0 ± 2.3	79.0 ± 1.8	JPYSG+AA	AA	24w	5.64 g	PSA, KPS
Tian SS^[11]^	2016	32	31	73.2 ± 9.5	72.8 ± 8.7	JZP+AA	AA	24w	5.4 g	PSA
Tang QC^[12]^	2018	17	17	71.24 ± 4.81	72.33 ± 3.16	JZP+AA	AA	24w	5.4 g	PSA
Li H^[13]^	2011	31	32	67.13 ± 7.53	68.72 ± 7.10	NCSC+AA	AA	12w	12 mL	Efficacy, PSA, adverse reaction
Chang DG^[14]^	2017	114	109	–	–	QLC+AA	AA	24w	20 mL	Efficacy
Chen DA^[37]^	2013	34	31	74.22 ± 5.94	73.71 ± 5.25	QLC+AA	AA	4w	250 mL	Efficacy, PSA
Zhang BH^[22]^	2007	11	6	–	–	BOE+AA	AA	12w	30 mL	PSA
Yang XH^[38]^	2011	31	30	–	–	BOE+AA	AA	24w	30 mL	PSA, Efficacy, adverse reaction
Chen J^[39]^	2013	29	29	75.97 ± 7.95	76.2l+8.32	BOE+AA	AA	12w	30ml	PSA, KPS, adverse reaction, Efficacy
Yu C^[40]^	2009	33	34	76.3 ± 4.2	77.6 ± 6.1	BOE+AA	AA	8w	30 mL	Efficacy, adverse reaction
Luo WC^[21]^	2018	44	44	76.87	76.24	BOE+AA	AA	8w	9 g	Efficacy
Su MY^[15]^	2013	30	30	70.46 ± 7.74	67.30 ± 11.39	ZBDHP	AA	4w	30 mL	Efficacy

AA = androgen antagonist, BOE = Bruceolic oil emulsion, C = control group, CKSI = Compound Kushen injection, I = intervention group, JPYSG = Jianpiyishen granule, JZP = Juzao pill, NCSC = Niaochangshu capsule, QLC = Qilan capsule, SFI = Shenfu injection, SQFZI = Shenqifuzheng injection, ZBDHP = Zhibodihuang pill.

### 3.3. Results of bias analysis and evaluation

**Figure 2.**
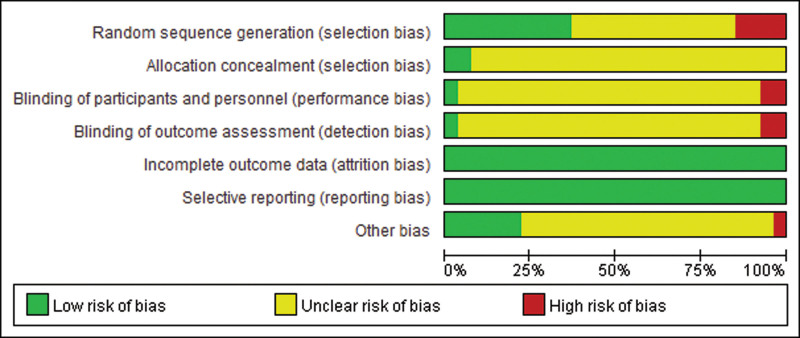
Results of risk of bias assessment.

In terms of randomization methods, 10 studies used the correct randomization method: 8 studies^[8–15]^ used the random number table method, and 2 studies^[16,17]^ used the envelope method. Four studies used incorrect randomization methods: one study^[18]^ by patient preference, 1 study^[19]^ by order of presentation, and 2 studies^[20,21]^ by intervention. The remaining studies did not indicate whether random methods were used. In terms of distribution hiding, 2 studies^[16,17]^ used the envelope method, and the other studies did not indicate whether distribution hiding was used. In terms of blind method, 1 study^[22]^ used blind method. Two studies^[11,12]^ knew the intervention in advance and were likely to be at high risk. In terms of completeness of results and selective reporting, all study results were complete and selective reporting was less likely to occur. In other aspects of bias. Six studies^[10–13,15,23]^ were less likely to have publication bias, 1 study^[22]^ had a small sample size and was more likely to have publication bias, and the other studies were unknown. Figure 2 shows the evaluation results of bias analysis.

### 3.4. Results of network meta-analysis

#### 3.4.1. Efficacy.

##### 3.4.1.1. Associations between interventions.

**Figure 3.**
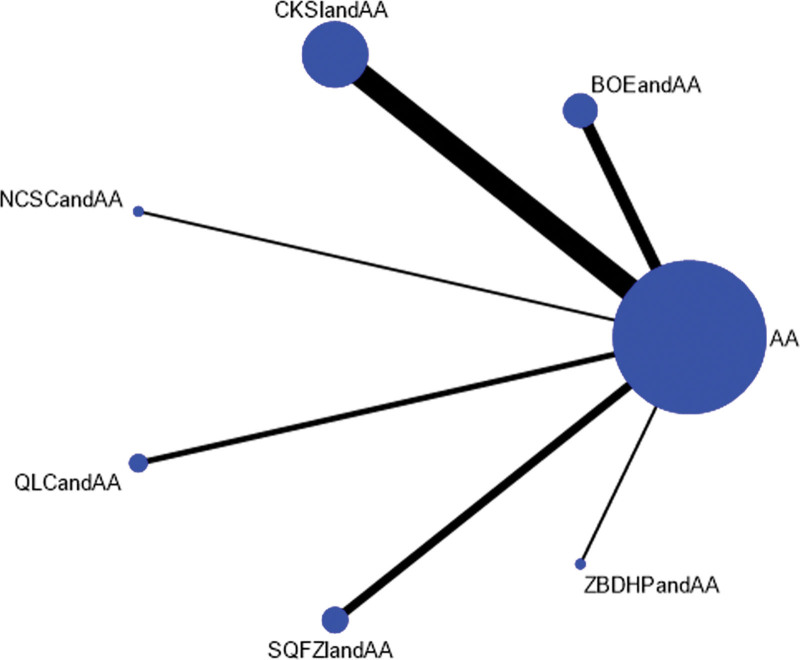
Network diagram of Chinese patent medicine in the treatment of efficacy as the outcome indicator.

Efficacy was reported in 19 studies, including 7 proprietary Chinese medicines. All studies only reported the comparison between proprietary Chinese medicines combined with androgen antagonists and androgen antagonists, and there was no direct pair-to-pair comparison between proprietary Chinese medicines. Among them, the largest number of studies have directly compared Compound Kushen injection combined with androgen antagonists to androgen antagonists (8 randomized controlled trials), graphically showing no closed loop (Fig. 3).

##### 3.4.1.2. Results of network meta-analysis.

**Figure 4.**
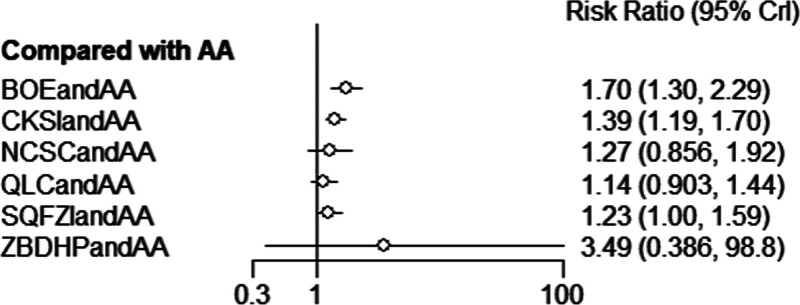
Forest diagram of Chinese patent medicine in the treatment of efficacy as the outcome indicator.

**Figure 5.**
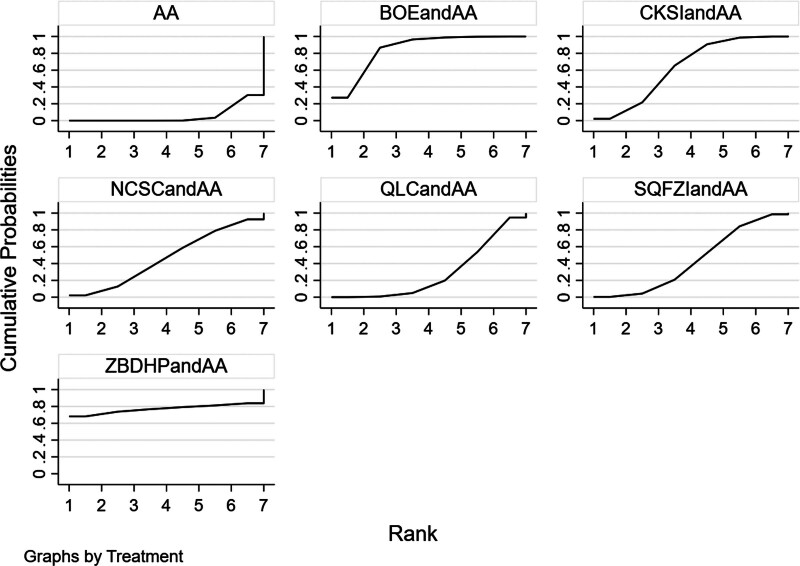
Efficacy SUCRA ranking results (larger area under the SUCRA curve suggests more effective interventions).

The results of network meta-analysis showed little overall heterogeneity (I^2^ = 7%). Compared with androgen antagonists, the 2 proprietary Chinese medicines combined with androgen antagonists had significant curative effects, which were Bruceolic oil emulsion (RR = 1.70, 95%CI [1.30, 2.29], *P* < .05). Compound Kushen injection (RR = 1.39,95%CI [1.19, 1.70], *P* < .05) (Fig. 4). There was no significant difference among all proprietary Chinese medicines (*P* > .05) (Table 2). The top 3 SUCRA rankings are: Bruceolic oil emulsion combined with androgen antagonist (83.2%), Zhibodihuang pill (80.4%), Compound Kushen injection combined with androgen antagonist (62.7%) (Table 6 and Fig. 5).

**Table 2:** League table (efficacy).

AA	BOEandAA	CKSIandAA	NCSCandAA	QLCandAA	SQFZIandAA	ZBDHPandAA
AA	**1.705 (1.287, 2.311**)	**1.394 (1.19, 1.707**)	1.265 (0.855, 1.94)	1.139 (0.903, 1.442)	**1.233 (1.002, 1.589**)	3.289 (0.386, 85.162)
0.587 (0.433, 0.777)	BOEandAA	0.82 (0.586, 1.155)	0.742 (0.451, 1.24)	0.668 (0.456, 0.959)	0.725 (0.502, 1.054)	1.924 (0.222, 50.817)
0.717 (0.586, 0.84)	1.22 (0.865, 1.705)	CKSIandAA	0.908 (0.574, 1.412)	0.818 (0.593, 1.068)	0.886 (0.66, 1.173)	2.352 (0.273, 59.868)
0.79 (0.515, 1.17)	1.348 (0.806, 2.218)	1.102 (0.708, 1.741)	NCSCandAA	0.901 (0.552, 1.423)	0.976 (0.609, 1.565)	2.603 (0.289, 68.526)
0.878 (0.694, 1.107)	1.498 (1.043, 2.193)	1.222 (0.936, 1.687)	1.11 (0.703, 1.81)	QLCandAA	1.083 (0.796, 1.532)	2.897 (0.334, 75.289)
0.811 (0.629, 0.998)	1.379 (0.949, 1.991)	1.128 (0.852, 1.515)	1.024 (0.639, 1.642)	0.923 (0.653, 1.256)	SQFZIandAA	2.657 (0.308, 68.876)
0.304 (0.012, 2.594)	0.52 (0.02, 4.506)	0.425 (0.017, 3.663)	0.384 (0.015, 3.465)	0.345 (0.013, 2.997)	0.376 (0.015, 3.246)	ZBDHPandAA

Bold values represent statistically significant results (*P* < 0.05).

#### 3.4.2. PSA.

##### 3.4.2.1. Associations between interventions.

**Figure 6.**
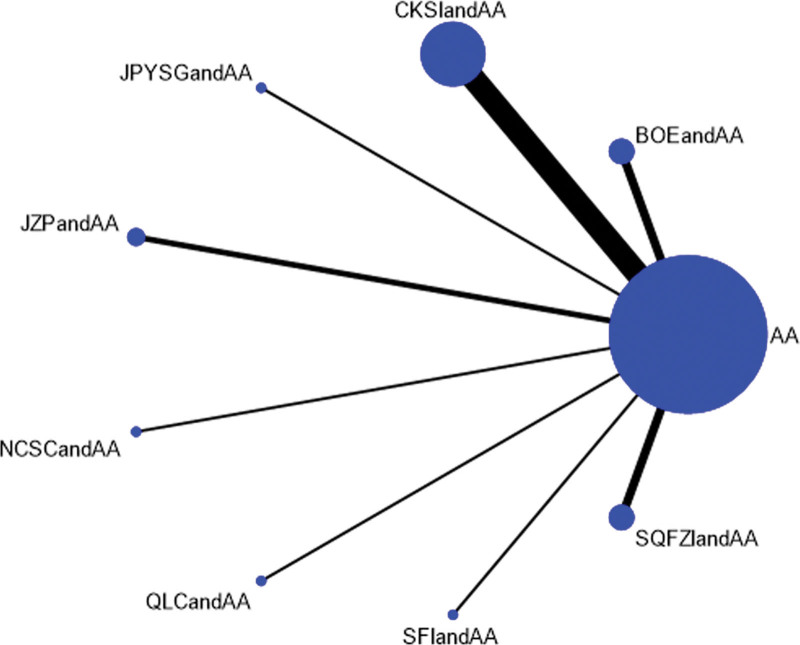
Network diagram of Chinese patent medicine in the treatment of PSA as the Outcome Indicator.

PSA levels were reported in 20 studies involving 8 proprietary Chinese medicines. All studies only reported the comparison between proprietary Chinese medicines combined with androgen antagonists and androgen antagonists, and there was no direct pair-to-pair comparison between proprietary Chinese medicines. Among them, the largest number of studies have directly compared Compound Kushen injection combined with androgen antagonists to androgen antagonists (8 randomized controlled trials), graphically showing no closed loop (Fig. 6).

##### 3.4.2.2. Results of network meta-analysis.

**Figure 7.**
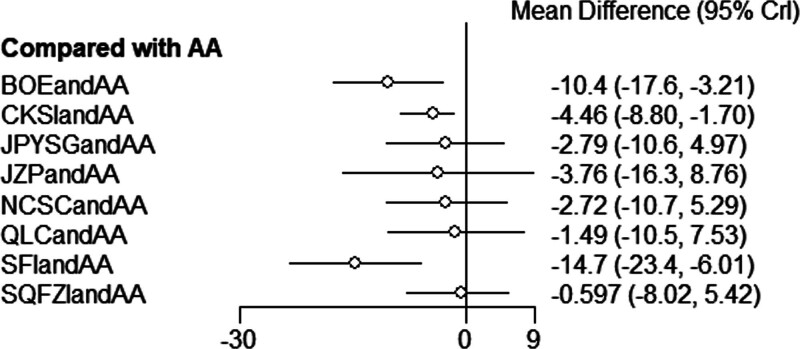
Forest diagram of Chinese patent medicine in the treatment of PSA as the outcome indicator.

**Figure 8.**
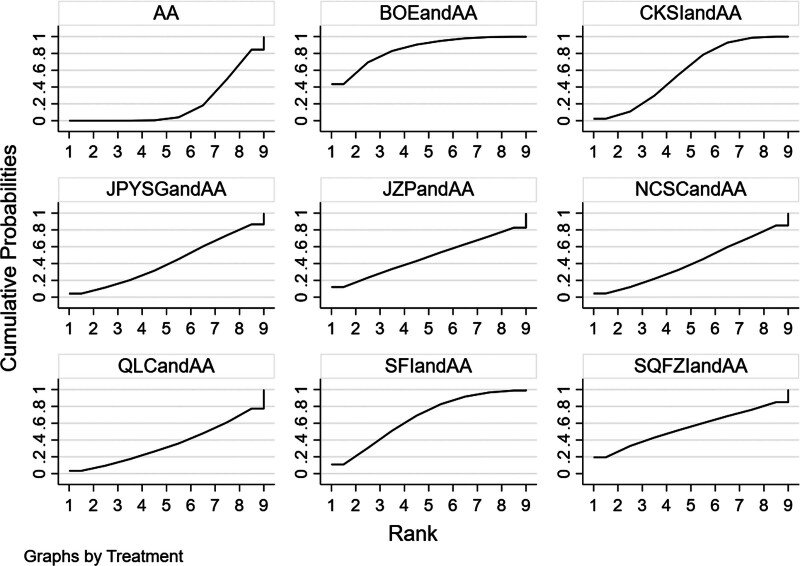
PSA SUCRA ranking results (larger area under the SUCRA curve suggests more effective interventions).

The results of network meta-analysis showed little overall heterogeneity (I2 = 0%). Compared with androgen antagonists, 3 proprietary Chinese medicines combined with androgen antagonists had significant curative effects, namely Bruceolic oil emulsion (MD = −10.4, 95%CI [−17.6, −3.21], *P* < .05). Compound Kushen injection (MD = −4.46, 95%CI [−8.80, −1.70], *P* < .05), compound Kushen injection (MD = −14.7, 95%CI [−23.4, −6.01], *P* < .05) (Fig. 7). There are also differences between proprietary Chinese medicines. The effect of Shenfu injection combined with androgen antagonist was better than CKSIandAA (MD = −10.26,95% CI [−18.95, −0.11], *P* < .05), Jianpi Yishen granule combined with androgen antagonist (MD = −11.95, 95% CI [−23.65, −0.26]. *P* < .05), Uichang Shu capsule combined with androgen antagonist (MD = −12.02, 95% CI [−23.87, −0.14], *P* < .05), Qilan capsule combined with androgen antagonist (MD = −13.25, 95%CI [−25.81, −0.68], *P* < .05). The effect of Shenfu injection combined with androgen antagonist was better than Shenqi Fuzheng injection combined with androgen antagonist (MD = −14.11, 95% CI (−24.37, −2.41), *P* < .05) (Table 3). The top 3 SUCRA rankings are: Shenfu injection combined with androgen antagonist (95.2%), Bruceolic oil emulsion combined with androgen antagonist (84.0%), Compound Kushen injection combined with androgen antagonist (58.5%) (Table 6 and Fig. 8).

**Table 3:** League table (PSA).

AA	BOEandAA	CKSIandAA	JPYSGandAA	JZPandAA	NCSCandAA	QLCandAA	SFIandAA	SQFZIandAA
AA	−**10.41** (−**17.71,** −**3.15**)	−**4.49** (−**8.82,** −**1.67**)	−2.78 (−10.6, 5.18)	−3.91 (−16.39, 8.65)	−2.72 (−10.72, 5.29)	−1.51 (−10.66, 7.6)	−**14.72** (−**23.52, -5.92**)	−0.62 (−8.08, 5.45)
10.41 (3.15, 17.71)	BOEandAA	5.87 (−2.8, 13.38)	7.62 (−3.07, 18.46)	6.48 (−7.81, 21.06)	7.67 (−3.06, 18.56)	8.89 (−2.79, 20.68)	−4.32 (−15.7, 7.14)	9.71 (−0.79, 19.17)
4.49 (1.67, 8.82)	−5.87 (−13.38, 2.8)	CKSIandAA	1.61 (−6.08, 11.22)	0.68 (−11.98, 14.15)	1.69 (−6.18, 11.35)	3 (−6.15, 13.51)	−10.22 (−18.99, 0.03)	3.77 (−3.58, 11.52)
2.78 (−5.18, 10.6)	−7.62 (−18.46, 3.07)	−1.61 (−11.22, 6.08)	JPYSGandAA	−1.16 (−15.68, 13.63)	0.05 (−11.23, 11.25)	1.28 (−10.77, 13.27)	−11.95 (−23.76, −0.22)	2.18 (−9.07, 11.8)
3.91 (−8.65, 16.39)	−6.48 (−21.06, 7.81)	−0.68 (−14.15, 11.98)	1.16 (−13.63, 15.68)	JZPandAA	1.23 (−13.69, 15.9)	2.41 (−13.19, 17.85)	−10.77 (−26.24, 4.31)	3.16 (−11.45, 16.91)
2.72 (−5.29, 10.72)	−7.67 (−18.56, 3.06)	−1.69 (−11.35, 6.18)	−0.05 (−11.25, 11.23)	−1.23 (−15.9, 13.69)	NCSCandAA	1.23 (−10.92, 13.37)	−**12.01** (−**23.9,** −**0.15**)	2.12 (−9.12, 11.79)
1.51 (−7.6, 10.66)	−8.89 (−20.68, 2.79)	−3 (−13.51, 6.15)	−1.28 (−13.27, 10.77)	−2.41 (−17.85, 13.19)	−1.23 (−13.37, 10.92)	QLCandAA	−**13.22** (−**25.91,** −**0.61**)	0.82 (−11.18, 11.58)
14.72 (5.92, 23.52)	4.32 (−7.14, 15.7)	10.22 (−0.03, 18.99)	11.95 (0.22, 23.76)	10.77 (−4.31, 26.24)	12.01 (0.15, 23.9)	13.22 (0.61, 25.91)	SFIandAA	**14.06 (2.36, 24.47**)
0.62 (−5.45, 8.08)	−9.71 (−19.17, 0.79)	−3.77 (−11.52, 3.58)	−2.18 (−11.8, 9.07)	−3.16 (−16.91, 11.45)	−2.12 (−11.79, 9.12)	−0.82 (−11.58, 11.18)	−14.06 (−24.47, −2.36)	SQFZIandAA

Bold values represent statistically significant results (*P* < 0.05).

#### 3.4.3. Adverse reaction.

##### 3.4.3.1. Associations between interventions.

**Figure 9.**
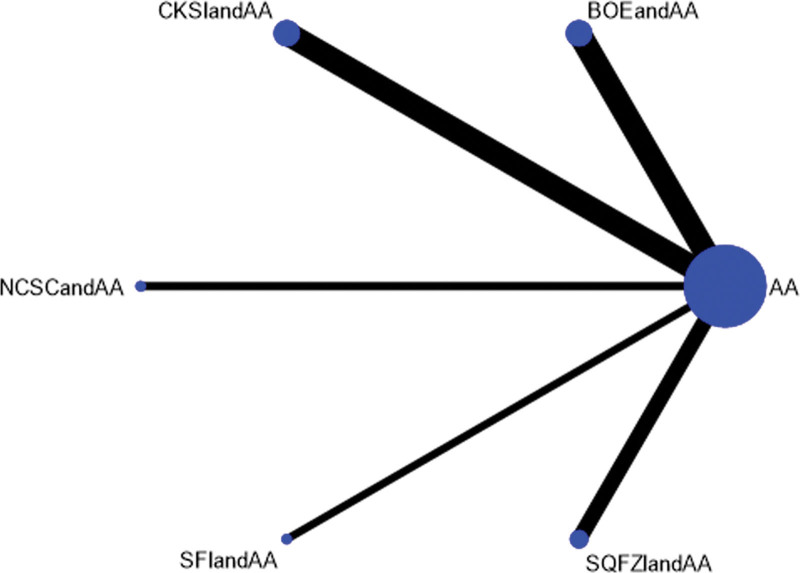
Network diagram of Chinese patent medicine in the treatment of adverse reaction as the outcome indicator.

Efficacy was reported in 10 studies, including 5 proprietary Chinese medicines. All studies only reported the comparison between proprietary Chinese medicines combined with androgen antagonists and androgen antagonists, and there was no direct pair-to-pair comparison between proprietary Chinese medicines. Among them, the largest number of studies have directly compared Compound Kushen injection combined with androgen antagonists to androgen antagonists (3 randomized controlled trials), graphically showing no closed loop (Fig. 9).

##### 3.4.3.2. Results of network meta-analysis.

**Figure 10.**
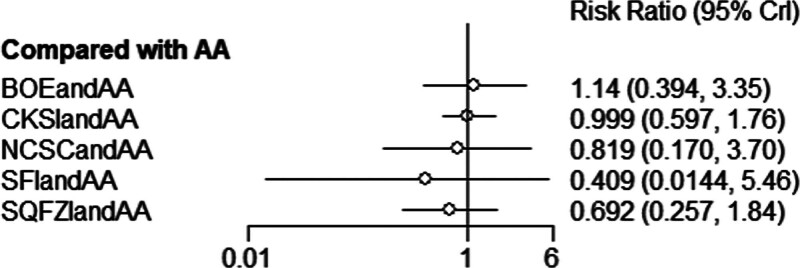
Forest diagram of Chinese patent medicine in the treatment of adverse reaction as the outcome indicator.

**Figure 11.**
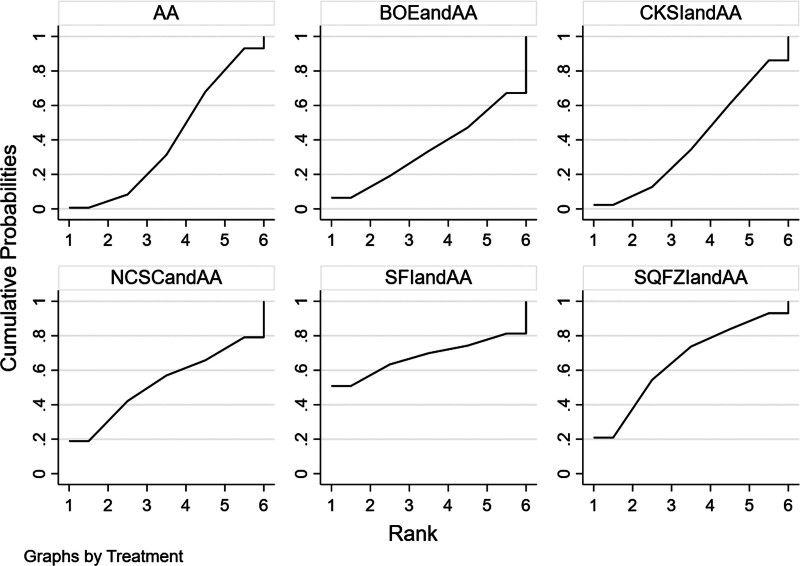
Adverse reaction SUCRA ranking results (larger area under the SUCRA curve suggests more effective interventions).

The results of network meta-analysis showed little overall heterogeneity (I2 = 0%). Compared with androgen antagonists, there was no significant difference among the proprietary Chinese medicines (*P* > .05) (Table 4 and Fig. 10). The top 3 SUCRA rankings are: Shenfu injection combined with androgen antagonist (67.5%), Shenqifuzheng injection combined with androgen antagonist (56.1%), Niaochangshu capsule combined with androgen antagonist (55.1%) (Table 5 and Fig. 11).

**Table 4:** League table (adverse reaction).

AA	BOEandAA	CKSIandAA	NCSCandAA	SFIandAA	SQFZIandAA
AA	1.123 (0.389, 3.281)	1.001 (0.598, 1.75)	0.806 (0.166, 3.631)	0.403 (0.014, 5.28)	0.689 (0.258, 1.829)
0.891 (0.305, 2.57)	BOEandAA	0.896 (0.274, 2.956)	0.716 (0.108, 4.568)	0.352 (0.011, 5.836)	0.612 (0.143, 2.577)
0.999 (0.571, 1.671)	1.116 (0.338, 3.654)	CKSIandAA	0.802 (0.149, 3.949)	0.399 (0.014, 5.514)	0.686 (0.222, 2.058)
1.241 (0.275, 6.022)	1.397 (0.219, 9.28)	1.248 (0.253, 6.71)	NCSCandAA	0.492 (0.013, 10.34)	0.855 (0.141, 5.421)
2.481 (0.189, 70.112)	2.838 (0.171, 90.769)	2.507 (0.181, 74.009)	2.032 (0.097, 76.097)	SFIandAA	1.733 (0.108, 53.753)
1.452 (0.547, 3.883)	1.633 (0.388, 6.988)	1.457 (0.486, 4.511)	1.169 (0.184, 7.097)	0.577 (0.019, 9.277)	SQFZIandAA

**Table5:** SUCRA sort (the larger the SUCRA value, the better the effect of the intervention).

	Efficacy		PSA		KPS		Adverse reaction	
	SUCRA	NO.	SUCRA	NO.	SUCRA	NO.	SUCRA	NO.
CKSI	62.7%	3	58.5%	3	61.3%	3	38%	6
NCSC	46.1%	5	42.7%	6	–	–	55.1%	3
QLC	28.7%	7	33.9%	7	–	–	–	–
SFI	57.8%	4	95.2%	1	34%	5	67.5%	1
SQFZI	42.6%	6	26.4%	8	64.9%	2	56.1%	2
BOE	83.2%	1	84.0%	2	43.2%	4	41.6%	5
ZBDHP	80.4%	2	–	–	–	–	–	–
JPYSG	–`	–	43.3%	5	73.9%	1	–	-
JZP	–	–	47.8%	4		–	–	–
AA	6.0%	8	17.8%	9	26.0%	6	48.5%	4

#### 3.4.4. Meta regression.

Since the included proprietary Chinese medicines had different course of treatment, meta regression was performed on the course of treatment and dose, and the influence of the course of treatment on the effect was discussed. The results showed no statistical significance (*P* < .05), indicating that compared with androgen antagonists, the clinical efficacy and safety of proprietary Chinese medicines were not significantly correlated with the duration and dose (Table 6).

**Table 6:** Results of meta regression.

Outcomes	Intervening measure	Regression coefficient (RR(95%CI))	
		Course	Dose
Efficacy	Compound Kushen injection	0.11 (−1.49, 1.72)	0.94 (−5.37, 8.02)
	Niaochangshu capsule	−0.57 (−31.82, 26.00)	1.48 (−20.86, 35.38)
	Qilan capsule	−0.37 (−2.74, 1.737)	−0.68 (−26.90, 19.84)
	Shenfu injection	0.45 (−21.80, 26.11)	−0.59 (−15.93, 13.75)
	Shenqifuzheng injection	−0.27 (3.51, 2.90)	−0.86 (−8.85, 11.85)
	Bruceolic oil emulsion	0.50 (−1.40, 2.42)	1.06 (−17.27, 26.92)
	Zhibodihuang pill	−0.79 (−22.78, 15.00)	−0.92 (−31.24, 28.52)
PSA	Compound Kushen injection	−1.10 (−16.56, 13.11)	−3.59 (-48.54, 43.85)
	Jianpiyishen granule	−2.16 (−74.23, 74.53)	3.95 (−49.75, 83.69)
	Juzao pill	−21.07 (−370.44, 133.89)	−3.65 (−139.41, 120.46)
	Niaochangshu capsule	12.33 (−390.28, 489.88)	−57.58 (−731.99, 114.59)
	Qilan capsule	0.04 (−107.97, 128.79)	4.70 (−165.67, 197.62)
	Shenfu injection	28.62 (−47.72, 91.87)	17.38 (−96.53, 82.93)
	Shenqifuzheng injection	4.60 (−31.32, 42.36)	2.47 (−30.57, 35.67)
	Bruceolic oil emulsion	1.76 (−35.04, 39.14)	−5.48 (−254.07, 197.85)
KPS	Compound Kushen injection	25.74 (−29.96, 60.94)	35.04 (−33.54, 121.63)
	Jianpiyishen granule	−52.65 (−306.75, 128.81)	3.68 (−204.67, 274.92)
	Shenfu injection	−153.80 (−1417.35, 127.11)	25.40 (−59.96, 115.70)
	Shenqifuzheng injection	1.85 (−206.82, 228.33)	−0.37 (−214.93, 180.24)
	Bruceolic oil emulsion	2.71 (−187.32, 214.66)	−13.86 (−315.88, 133.69)
Adverse reaction	Compound Kushen injection	2.88 (−2.87, 11.86)	1.58 (−8.43, 21.02)
	Niaochangshu capsule	−2.13 (−35.92, 23.00)	3.23 (−17.74, 67.97)
	Shenfu injection	−0.15 (−21.15, 19.15)	−0.06 (−26.42, 24.38)
	Shenqifuzheng injection	−0.46 (−3.71, 2.51)	−0.35 (−2.61, 1.84)
	Bruceolic oil emulsion	−0.03 (−2.70, 2.54)	−0.15 (−22.04, 19.02)

#### 3.4.5. Secondary outcome indicators.

**Figure 12.**
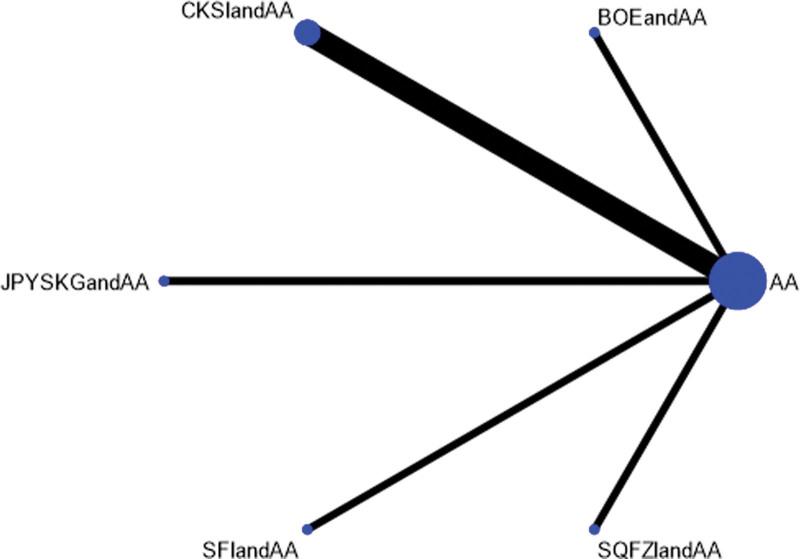
Network diagram of Chinese patent medicine in the treatment of KPS as the outcome indicator.

**Figure 13.**
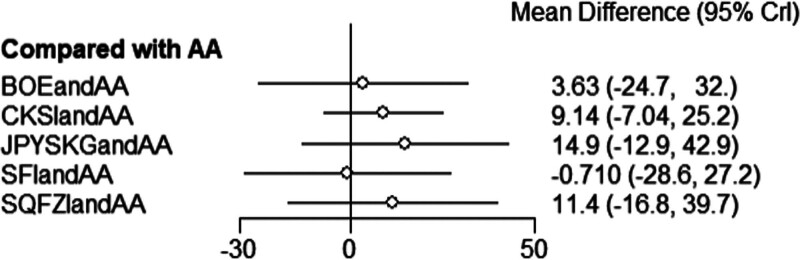
Forest diagram of Chinese patent medicine in the treatment of KPS as the outcome indicator.

**Figure 14.**
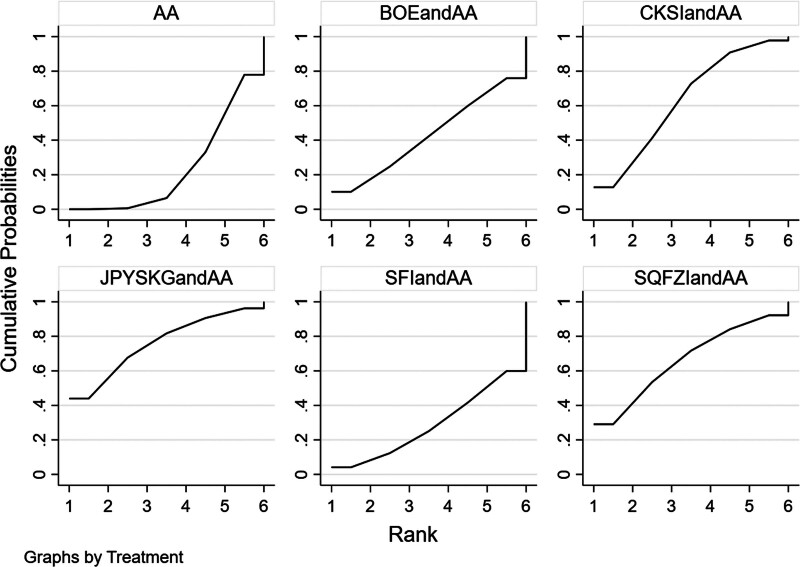
KPS SUCRA ranking results (larger area under the SUCRA curve suggests more effective interventions).

Efficacy was reported in 7 studies, including 5 proprietary Chinese medicines. All studies only reported the comparison between proprietary Chinese medicines combined with androgen antagonists and androgen antagonists, and there was no direct pair-to-pair comparison between proprietary Chinese medicines. Among them, Compound Kushen injection combined with androgen antagonists has the most studies directly compared with androgen antagonists (3 randomized controlled trials), graphically showing no closed loop. The results of Network Meta-analysis showed little overall heterogeneity (I2 = 7%). Compared with androgen antagonists, there was no significant difference among all proprietary Chinese medicines (*P* > .05). The top 3 SUCRA rankings are: Jianpiyishen granule combined with androgen antagonist (73.9%), Shenqifuzheng injection combined with androgen antagonist (64.9%), Compound Kushen injection combined with androgen antagonists (61.3%) (Appendix S1, Supplemental Digital Content, http://links.lww.com/MD/L588, Figs. 12–14, Table 7).

**Table 7:** League table (KPS).

AA	BOEandAA	CKSIandAA	JPYSKGandAA	SFIandAA	SQFZIandAA
AA	3.71 (−24.45, 31.69)	9.17 (−6.74, 25.12)	14.98 (−12.65, 42.48)	−0.66 (−28.36, 27.04)	11.52 (−16.52, 39.58)
−3.71 (−31.69, 24.45)	BOEandAA	5.46 (-−6.78, 37.72)	11.28 (−28.18, 50.61)	−4.39 (−43.8, 35.11)	7.79 (−31.93, 47.47)
−9.17 (−25.12, 6.74)	−5.46 (−37.72, 26.78)	CKSIandAA	5.77 (26.28, 37.65)	−9.83 (−41.96, 21.96)	2.32 (29.85, 34.58)
−14.98 (−42.48, 12.65)	−11.28 (−50.61, 28.18)	−5.77 (−37.65, 26.28)	JPYSKGandAA	−15.67 (−54.7, 23.42)	−3.42 (−42.73, 35.86)
0.66 (−27.04, 28.36)	4.39 (−35.11, 43.8)	9.83 (−21.96, 41.96)	15.67 (−23.42, 54.7)	SFIandAA	12.21 (-27.16, 51.59)
−11.52 (-39.58, 16.52)	−7.79 (−47.47, 31.93)	−2.32 (−34.58, 29.85)	3.42 (−35.86, 42.73)	−12.21 (−51.59, 27.16)	SQFZIandAA

#### 3.4.6. Publication bias assessment.

**Figure 15.**
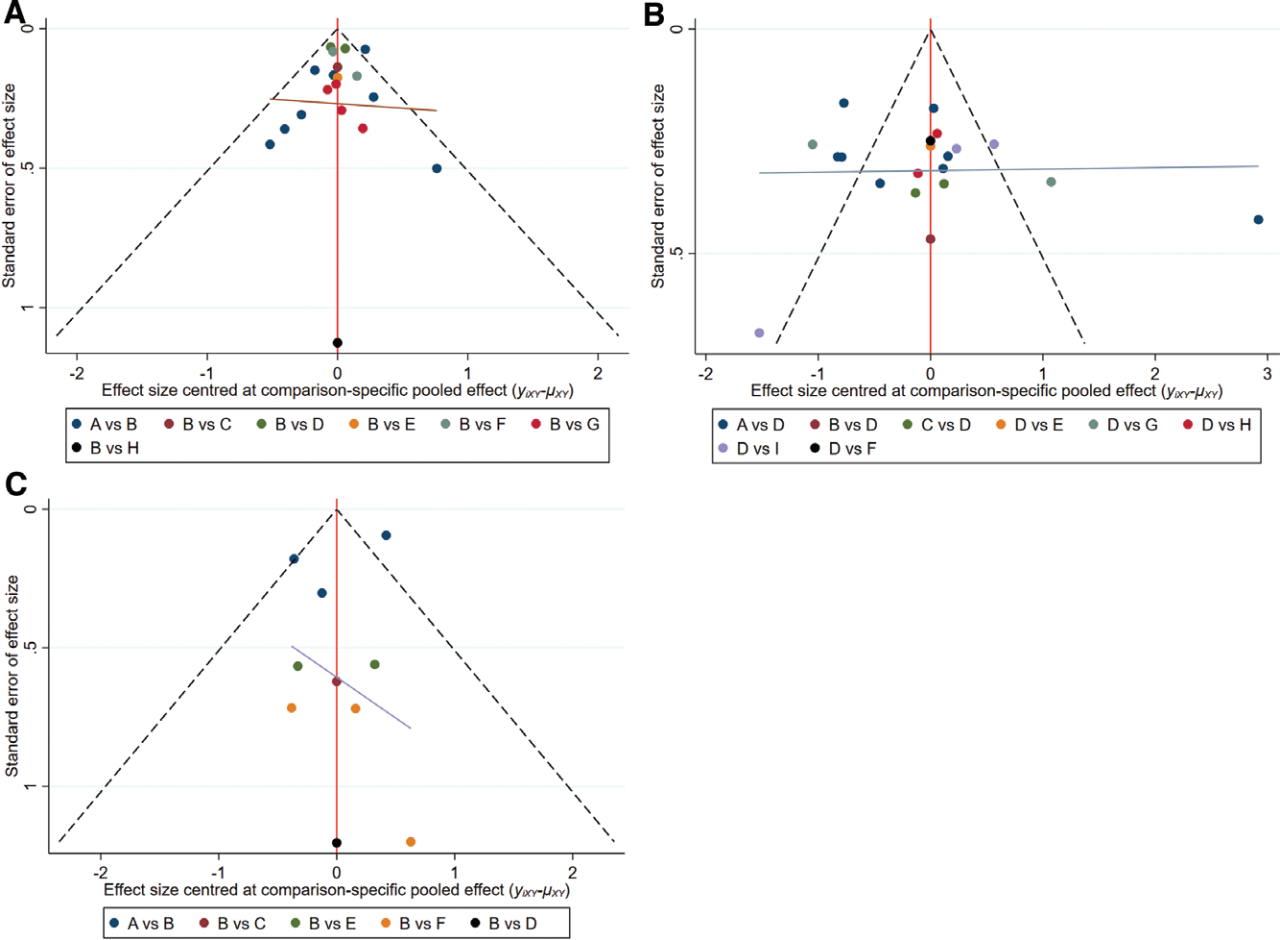
(A) Comparison-corrected funnel plot (efficacy). (B) Comparison-corrected funnel plot (PSA). (C) Comparison-corrected funnel plot (adverse reaction).

Efficacy, serum PSA level, and adverse reaction were evaluated for publication bias, and a comparation-correction funnel diagram was prepared. The results show that points in the comparation-correction funnel diagram are basically symmetrical in the middle line, indicating that publication bias is less likely to occur (Fig. 15A–C).

## 4. Discussion

This study included 27 studies, including 9 proprietary Chinese medicines: Shenfu injection, Shenqifuzheng injection, Compound Kushen injection, Jianpiyishen granule, Juzao pill, Niaochangshu capsule, Qilan capsule, Brucea oil emulsion, Zhibodihuang pill. A total of 4 main outcome measures were studied. In terms of Efficacy, Compound Kushen injection combined with androgen antagonists and Bruceolic oil emulsion combined with androgen antagonists have significant advantages compared with androgen antagonists. Compound Kushen injection combined with androgen antagonist and Bruceolic oil emulsion combined with androgen antagonist have significant advantages in reducing serum PSA level. Compared with androgen antagonists, Chinese proprietary drugs combined with androgen antagonists showed no advantage in improving quality of life score. In terms of safety, each proprietary Chinese medicine combined with androgen antagonist showed no advantage. adverse reaction of PMS combined with androgen antagonists is usually adverse reaction including hypolibido, fever, musculoskeletal pain, allergy, and gastrointestinal reaction. The symptoms are relatively mild. Studies have reported that patients can generally relieve the adverse reaction on their own or recover after treatment. Meta regression results showed that the effect of Chinese patent medicine combined with androgen antagonists was less affected by the good dose of treatment. In terms of specific outcome indicators, SUCRA sequencing results showed that different proprietary Chinese medicines had different effects and each had its own advantages, but the difference between different proprietary Chinese medicines was not significant. Based on comprehensive analysis, Bruceolic oil emulsion combined with androgen antagonist is the best intervention measure, which can improve the reference for the clinical use of proprietary Chinese medicine in the treatment of prostate cancer, and should be considered in clinical use.

Bruceolic oil emulsion is an extract from the fruit of Brucea Javanica(L)Merr. Pharmacological studies show that Bruceolic BJ has strong anticancer activity on various types of cancer cells, such as lung, colorectal, pancreas, stomach and leukemia cells. Its main mechanisms are inhibition of cell proliferation, induction of apoptosis, inhibition of cell migration and invasion, induction of autophagy and inhibition of angiogenesis.^[24]^ Many ingredients in BJ have anticancer effects. In particular, Brusatol is a bioactive ingredient of particular note. Brusatol has been shown in many studies to be a potent inhibitor of Nrf2, reducing the protein levels of Nrf2 in different cancer cells. It can inhibit cell proliferation and metastasis and induce cell cycle arrest and apoptosis.^[24]^ It can also be combined with chemotherapy, radiation therapy and phototherapy to achieve better anti-cancer effects, while reducing chemotherapy resistance and increasing the sensitivity of cancer cells to many traditional anti-cancer drugs.^[25]^ At the same time, Brustol did not cause significant toxic side effects in vivo,^[26]^ which has the potential to develop anticancer drugs.

Bruceolic oil emulsion is an oil-in-water emulsion made from the fat oil extracted from dried ripe fruits by emulsification. The oleic acid contained in the membrane has a specific affinity, which can destroy the membrane systems of PC3 cells of prostate cancer, such as the endoplasmic membrane, mitochondrial membrane and nuclear membrane, thereby killing prostate cancer cells.^[27]^ It can also change the cycle of prostate cancer cells by inhibiting topoisomerase (TOPO11) activity, make a large number of prostate cancer cells gather in the G0/ G1 phase, do not enter the S phase, inhibit the DNA synthesis of prostate cancer cells, and block the growth process of prostate cancer cells. The oil granules in Brucea javanea oil emulsion have specific affinity, which can adhere to cancer cells for a long time, penetrate into cancer cells, and improve anti-tumor effect.^[28]^ Brucea oil milk also has the effect of improving immunity. The particle diameter of Brucea javanica emulsion is <5m, it can pass through the blood brain barrier through the capillary wall, and has a high concentration in the liver, spleen, kidney, lung and other organs, which can promote the hematopoietic function of the bone marrow, improve the body immunity, and have the effect of strengthening and strengthening the root.

At present, traditional meta-analyses of proprietary Chinese medicines in the treatment of prostate cancer have been published. The results of the study by Yu Meili et al^[29]^ showed that Compound Kushen injection combined with androgen antagonist had a better effect than single androgen antagonist in reducing serum PSA level (MD = −0.40, 95%CI (−0.51, −0.29), *P* < .00001). Brucea javanica injection combined with androgen antagonist was superior to single androgen antagonist (MD = −9.29, 95% CI (−11.63, −6.95), *P* < .05), which was consistent with the results of this study. However, his study found that Kushen injection and Shenqifuzheng injection combined with androgen antagonists were superior to simple androgen antagonists. This is inconsistent with the results of this study. In terms of safety, Yu Meili et al found that Kushen injection, Shenqifuzheng injection combined with androgen antagonists were superior to simple androgen antagonists. The analysis showed that the number of literatures included in these 2 interventions was small (1 article for matrine injection and 1 article for Shenqifuzheng Injection). This may make the results less convincing. Further clinical studies are needed.

## 5. Advantages and limitations of this study

Advantages of this study: For the first time, we conducted a Network Meta-analysis of proprietary Chinese medicines for prostate cancer treatment, comparing the effectiveness and safety differences of different proprietary Chinese medicines combined with androgen antagonists. At the same time, the influence of treatment course on efficacy and safety was considered. In addition, we used strict inclusion and exclusion criteria, including only full-text randomized controlled trials.

Limitations of this study: The number of included studies was small, and there were differences in the selection of efficacy evaluation criteria, patient characteristics, sample size, and outcome indicators. Some studies are not stated in terms of randomized methods, assignment concealment, blind methods, and these factors may have some impact on the strength of evidence; Most of the included literature described TCM syndrome types, which may reduce the scientific results. English literature was not included, which may have the possibility of publication bias. Some outcome indicators selected in this study included a small amount of original literature, which may also have a certain impact on the results. Due to the lack of follow-up, key outcome indicators such as survival time and survival rate could not be obtained, which reduced the reliability of results. Therefore, more large, multi-center, high-quality randomized controlled trials are needed to provide more evidence in the future.

## 6. Conclusion

Based on the results of this study, compared with androgen antagonists, Compound Kushen injection combined with androgen antagonists and Bruceolic oil emulsion combined with androgen antagonists have significant advantages. The effects of different proprietary Chinese medicines are different. In summary, Bruceolic oil emulsion may be the best proprietary Chinese medicine, Bruceolic oil emulsion combined with androgen antagonists may be the best intervention measures, which can be used as a reference for clinical use of proprietary Chinese medicines in the treatment of prostate cancer. In clinical practice, corresponding treatment measures should be given according to different conditions of patients.

## Acknowledgments

We would like to thank the researchers and study participants for their contributions.

## Author contributions

**Funding acquisition:** Peijie Qin.

**Supervision:** Jianfeng Liu.

**Writing – review & editing:** Shiheng Wang, Fengxia Zhang.

## Supplementary Material


