# Supplementary material for: Efficacy and safety of Chinese patent medicine in the adjuvant treatment of prostate cancer: A Bayesian network meta-analysis
Source: Medicine (Baltimore). 2024 Mar 22;103(12):e37180. doi: 10.1097/MD.0000000000037180 (PMC10956989; doi:10.1097/MD.0000000000037180)
Supplement: Supplementary file 1 [file medi-103-e37180-s001.pdf]

KPS

(1) Associations between interventions

KPS was reported in seven studies, including five proprietary Chinese medicines. All studies only reported the comparison between proprietary Chinese medicines combined with androgen antagonists and androgen antagonists, and there was no direct pair-to-pair comparison between proprietary Chinese medicines. Among them, the largest number of studies have directly compared Compound Kushen injection combined with androgen antagonists to androgen antagonists (3 randomized controlled trials), graphically showing no closed loop (Figure 12).

(2) Results of mesh meta-analysis

The results of mesh meta-analysis showed little overall heterogeneity (I2=7%). Compared with androgen antagonists, there was no significant difference among proprietary Chinese medicines ($P > 0.05$) (Table 7, Figure13). The top three SUCRA rankings are: Jianpiyishen granule combined with androgen antagonist (73.9%), Shenqifuzheng injection combined with androgen antagonist (64.9%), Compound Kushen injection combined with androgen antagonist (61.3%) (Table 7, Figure14).
